# Typhus Fever: An Overlooked Diagnosis

**DOI:** 10.3329/jhpn.v27i3.3385

**Published:** 2009-06

**Authors:** Ramendra N. Mazumder, Mark A.C. Pietroni, Nadira Mosabbir, M.A. Salam

**Affiliations:** ^1^ Dhaka Hospital, ICDDR,B, GPO Box 128, Dhaka 1000, Bangladesh; ^2^ Clinical Sciences Division, ICDDR,B, GPO Box 128, Dhaka 1000, Bangladesh

**Keywords:** Co-infections, Morbidity, Typhoid, Typhus, Bangladesh

## Abstract

A case of typhus fever is presented. On admission, the clinical diagnosis was typhoid fever. Forty-eight hours after admission, the presence of subconjunctival haemorrhage, malena, and jaundice raised the possibility of a different aetiology, the two most likely differentials being dengue and typhus. Finally, a co-infection of typhoid and typhus was discovered. This uncommon clinical scenario should be taken into account in the management of patients with high fever on admission being treated as a case of typhoid fever.

## INTRODUCTION

Epidemic and endemic typhus is sometimes misdiagnosed as typhoid fever in tropical countries. Similarly, co-infection of typhoid and typhus fever can be overlooked if not suspected clinically. High continued fever with variable associated symptoms, such as malaise, headache, and myalgia, are usually present in both typhoid and typhus fever. Jaundice and malena may also be present in some cases of both typhoid and typhus fever. However, signs such as subconjunctival haemorrhage should lead to a different aetiology of fever being considered, including typhus fever. Therefore, clinical suspicion is of paramount importance in the diagnosis of both typhoid and typhus fever. Confirmation of the diagnosis is important as the treatment is different but may not always be possible. In most cases of typhus, treatment is given based on clinical suspicion or a positive Weil-Felix test. In clinically-suspected cases of typhus fever, a rising titre of OX_K_, OX2, and OX19 antigens supports the diagnosis but confirmation of the diagnosis may be difficult. Isolation of *Salmonella* Typhi from blood confirms the diagnosis of typhoid fever. Here, we present a case of typhoid fever with a co-infection of typhus fever in a patient recently admitted to the Dhaka Hospital of ICDDR,B.

## HISTORY

In February 2008, a previously-healthy 20-year old adult labourer with complaints of acute watery diarrhoea and high continued fever for five days was admitted to the Longer Stay Unit of the Dhaka Hospital of ICDDR,B. He received unspecified medicines at home. No significant past illness was reported.

On admission, the patient was alert and oriented with high fever (40 °C); no pallor, jaundice, or cyanosis was noted. His radial pulse was 120 bpm, regular and good in volume; respiration rate was 24 per minute; and BP was 100/50 mm Hg.

On examination, his breath sounds were vesicular, with no added sounds. Abdomen was soft and non-tender, and bowel sounds were active. Liver and spleen were not palpable. Other systemic examination revealed no abnormality.

His problems were listed as: (a) acute watery diarrhoea and (b) fever.

Clinical impression was enteric fever with a differential of viral fever.

Random blood glucose on admission was 9.0 mmol/L. Complete blood count, blood for culture, and rectal swab for culture were requested, and intravenous (IV) ceftriaxone was started.

Laboratory investigation showed that total white blood-cell count (TWBC) was 3,000/mm^3^ with polymorphs–80%, band–4%, lymp–14%, and monocyte–2%.

After 24 hours, the patient was found to be toxic, highly febrile, and developed conjunctival injection (subconjunctival haemorrhage) (Fig.). So, a differential of dengue and typhus fever was considered.

**Fig.**
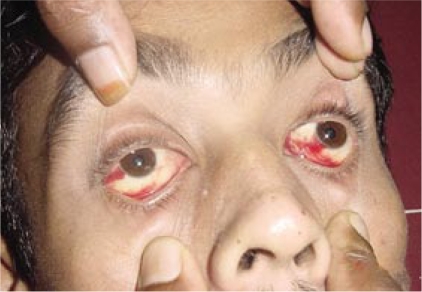
Conjunctival suffusion

After two days, the patient complained of passing black stool, so a repeat blood count with platelets, stool M/E, dengue ELISA IgM, and IgG was performed.

Repeat TWBC was 3,500/mm^3^, haematocrit 31%, and platelets 2,25,000/mm^3^.

Stool examination showed: red blood-cells: 1-5/high power field (HPF), pus cells: 6-10/HPF, and occult blood test was positive.

Dengue ELISA: Ig M–negative and IgG–positive, indicative of a past infection.

Till the 5^th^ day of admission, blood culture revealed no growth. Widal titres were performed: TH was 1:160, and others were 1:20.

Weil-Felix titres were: OX2=1:80, OX19=1:160, and OXk=1:160

On the 6th day, the patient was found to be icteric with no hepato-spleenomegaly. Liver function tests were performed: serum bilirubin–6.7 µmol/L, S. ALT–113 U/L, S. AST–98 U/L, and S. ALP–101 U/L. Blood culture grew *S.* Typhi which was sensitive to ceftriaxone, intermediately sensitive to ciprofloxacin, and resistant to amoxycillin.

After 10 days, repeat stool microscopy revealed no red blood-cells. Subconjuctival haemorrhage regressed. The patient was afebrile on day 12 after admission and was discharged 14 days after admission. He was advised to come for follow-up after seven days to assess the need to treat for typhus fever (which had been withheld due to his raised liver transaminases).

## DISCUSSION

Typhus is caused by rickettsial organisms, a group of Gram-negative coccobacilli and short bacilli and results in an acute febrile illness. Arthropod vectors transfer aetiologic agents to humans. Various types of rickettsial diseases have been described; of them, (a) epidemic/louse-borne typhus is caused by *Rickettsia prowazekii,* and the vector is the body-louse. It has a worldwide distribution; (b) Murine typhus is caused by *R. typhi,* and the vector is the rat or cat flea (*Xenopsylla cheopis, Ctenocephalides felis*). It has also a worldwide distribution; and (c) Scrub typhus is caused by *Orientia tsutsugamushi* (formerly *Rickettsia tsutsugamushi*) transmitted via the mite belonging to the *Leptotrombidium akamushi,* and possibly *Leptotrombidium deliense* (1)*.* It is common in Asia, Australia, Papua New Guinea, and Pacific islands.

History of exposure, bite by vectors, fever, headche, rash (RMSF), regional lymphadenopathy are the most common presentations. High fever (∼40 °C), lymphadenopathy, Eschar-painless papule, hepatomegaly, spleenomegaly, and conjunctival suffusion are commonly present.

There are reports of typhus fever from Bangladesh (2).

### Pathophysiology

Scratching a louse-bite site allows the rickettsia-laden excrement to be inoculated into the bite wound. The rickettsia travel to the bloodstream, and rickettsemia develops. The major pathology is caused by a vasculitis and its complications.

Common differentials are: dengue, malaria, typhoid fever, brucellosis, and infectious mononucleosis.

Laboratory investigations are not particularly helpful; however, they can assist in assessing the severity of the illness and help in excluding other diseases from the differential diagnosis.

Leukopenia is common in the early stages of disease. Total white cell count may be normal or mildly elevated. Thrombocytopenia is common.

A rise in IgM titre indicates an acute primary disease, and IgG indicates past infection. A rise in OX titre is suggestive of typhus fever, and polymerase chain reaction (PCR) for typhus is confirmatory.

Diagnosis is mostly clinical: history of travel, cold weather or crowded environment, high fever, skin-rash, conjunctival haemorrhage and/or gastrointestinal haemorhage are supportive of the diagnosis. Weil-Felix test is considered sufficient for diagnosis in most cases but PCR is confirmatory (1,2).

Most cases are treated with doxycycline (100 mg PO bid for 5 days) or cholramphenicol (500 mg qid PO for 7-10 days) or ciprofloxacin (750 mg bid PO for 5 days). Mortality from untreated typhus fever is up to 15% (3). In this case, we treated the patient with ceftriaxone for typhoid fever. We did not treat the patient with doxycycline, chloramphenicol, or ciprofloxacin because of high liver enzymes but monitored for any clinical deterioration of symptoms from typhus. The patient was asked to return one week after discharge to receive a second course of antibiotics for typhus.

Most common complications are due to vasculitis: hepatitis, gastrointestinal haemorrhage, hypovolaemia, electrolyte imbalance, multi-organ involvement, including CNS and kedneys, have been reported. Secondary infection such as pneumonia is not uncommon.

The disease can be prevented by maintaining good personal hygiene, use of insecticides to reduce rodent population, and by avoiding exposure in endemic areas.
